# Respiratory FimA-Specific Secretory IgA Antibodies Upregulated by DC-Targeting Nasal Double DNA Adjuvant Are Essential for Elimination of *Porphyromonas gingivalis*

**DOI:** 10.3389/fimmu.2021.634923

**Published:** 2021-02-25

**Authors:** Kosuke Kataoka, Shigetada Kawabata, Kayo Koyanagi, Yoshiya Hashimoto, Tatsuro Miyake, Kohtaro Fujihashi

**Affiliations:** ^1^ Department of Preventive and Community Dentistry, Faculty of Dentistry, Osaka Dental University, Hirakata, Japan; ^2^ Department of Oral and Molecular Microbiology, Graduate School of Dentistry, Osaka University, Suita, Japan; ^3^ Department of Biomaterials, Faculty of Dentistry, Osaka Dental University, Hirakata, Japan; ^4^ Division of Clinical Vaccinology, International Research and Development Center for Mucosal Vaccines, The Institute of Medical Science, The University of Tokyo, Tokyo, Japan; ^5^ Department of Pediatric Dentistry, School of Dentistry, The University of Alabama at Birmingham, Birmingham, AL, United States

**Keywords:** nasal vaccine, recombinant FimA (*r*FimA), dendritic cells (DCs), double DNA adjuvant, mucosal IgA, *Porphyromonas gingivalis*

## Abstract

Our previous studies showed that a combination of a DNA plasmid encoding Flt3 ligand (pFL) and CpG oligodeoxynucleotides 1826 (CpG ODN) (FL/CpG) as a nasal adjuvant provoked antigen-specific immune responses. In this study, we investigated the efficacy of a nasal vaccine consisting of FimA as the structural subunit of *Porphyromonas gingivalis* (*P. gingivalis*) fimbriae and FL/CpG for the induction of FimA-specific antibody (Ab) responses and their protective roles against nasal and lung infection by *P. gingivalis*, a keystone pathogen in the etiology of periodontal disease. C57BL/6 mice were nasally immunized with recombinant FimA (*r*FimA) plus FL/CpG three times at weekly intervals. As a control, mice were given nasal *r*FimA alone. Nasal washes (NWs) and bronchoalveolar lavage fluid (BALF) of mice given nasal *r*FimA plus FL/CpG resulted in increased levels of *r*FimA-specific secretory IgA (SIgA) and IgG Ab responses when compared with those in controls. Significantly increased numbers of CD8- or CD11b-expressing mature-type dendritic cells (DCs) were detected in the respiratory inductive and effector tissues of mice given *r*FimA plus FL/CpG. Additionally, significantly upregulated Th1/Th2-type cytokine responses by *r*FimA-stimulated CD4^+^ T cells were noted in the respiratory effector tissues. When mice were challenged with live *P. gingivalis via* the nasal route, mice immunized nasally with *r*FimA plus FL/CpG inhibited *P. gingivalis* colonization in the nasal cavities and lungs. In contrast, controls failed to show protection. Of interest, when IgA-deficient mice given nasal *r*FimA plus FL/CpG were challenged with nasal *P. gingivalis*, the inhibition of bacterial colonization in the respiratory tracts was not seen. Taken together, these results show that nasal FL/CpG effectively enhanced DCs and provided balanced Th1- and Th2-type cytokine response-mediated *r*FimA-specific IgA protective immunity in the respiratory tract against *P. gingivalis.* A nasal administration with *r*FimA and FL/CpG could be a candidate for potent mucosal vaccines for the elimination of inhaled *P. gingivalis* in periodontal patients.

## Introduction

Secretory IgA (SIgA) antibody (Ab) is the major isotype at the mucosal surface. SIgA Abs are mainly secreted as dimeric or polymeric forms and play roles as the first line of defense by neutralizing viruses and toxins as well as by inhibiting bacterial adherence to host mucosal surfaces (1). Hence, mucosal immunization can be a major strategy for the induction of antigen (Ag)-specific SIgA Ab responses (1). For example, nasal vaccination effectively induces Ag-specific SIgA Ab responses in various mucosal tissues including the respiratory tract (2), the oral cavity (3), and the reproductive tract (4). An additional unique feature of mucosal immunization is to elicit Ag-specific IgG Ab responses in the systemic compartment (2–5). Despite these advantages, mucosal immunization requires adjuvants or a delivery system for the induction and regulation of Ag-specific immune responses. In this regard, we have previously shown that nasal application of a DNA plasmid encoding Flt3 ligand cDNA (pFL) as a mucosal adjuvant and ovalbumin as an Ag preferentially expands CD8^+^ CD11c^+^ dendritic cells (DCs) and subsequently induces IL-4-producing CD4^+^ T cell-mediated Ag-specific mucosal immune responses (6). Further, we have shown that the combination of pFL and CpG oligodeoxynucleotides (FL/CpG) as a DC-targeting nasal adjuvant enhances Ag-specific mucosal and systemic immunity with a balanced Th1/Th2-type cytokine response that protects from bacterial and viral infection (7–9).

Periodontitis is one of the most prevalent infectious diseases worldwide and is characterized by gingival inflammation and bone loss following periodontal-pathogenic bacterial infection and disruption of host immunity. *Porphyromonas gingivalis* (*P. gingivalis*) is a keystone pathogen that is responsible for the progression of periodontitis (10) and various systemic diseases including aspiration pneumonia (11), despite being detected in healthy people (12). Fimbriae on the cell surfaces of *P. gingivalis* are known to be adhesins primarily composed of polymers of FimA protein (fimbrillin) encoded by the gene *fimA* (13). Thus, this protein is considered to be a virulence factor and plays an important role in initial attachment or colonization through its association with salivary proteins and other bacteria on the surfaces of oral mucosa or teeth (14, 15). For example, it has been reported that fimA-inactivated mutants have lessened the ability to adhere to human gingival fibroblasts and epithelial cells (16). In addition, it has also been proven that the FimA protein elicits inflammatory responses *via* the TLR4/NF-κB signaling pathway in human peripheral blood mononuclear cells (17). Our previous study also showed that recombinant FimA (*r*FimA) protein specifically and rigidly binds to human salivary proteins such as salivary statherin or proline-rich protein (15).

In this study, we investigated the efficacy of a nasal vaccine consisting of FimA and FL/CpG for the induction of FimA-specific Ab responses and their functional properties against nasal and pulmonary infection with *P. gingivalis.* Our results showed an essential role of *r*FimA-specific SIgA Abs for the prevention of *P. gingivalis* colonization in the upper and lower airways.

## Materials and Methods

### DNA Adjuvants

The plasmid pUNO1-mFlt3L (pFL) consists of the pUNO1-mcs vector plus the full-length murine FL cDNA gene (InvivoGen, San Diego, CA, USA). This plasmid was purified using the EndoFree Plasmid purification Giga kit (QIAGEN, Valencia, CA, USA). The Limulus amebocyte lysate assay (BioWhittaker, Walkersville, MD, USA) resulted in <0.1 endotoxin units of LPS per 1 µg of plasmid. A synthetic ODN containing CpG motif 1826 (CpG ODN) (FASMAC Co., Ltd., Kanagawa, Japan) was synthesized artificially.

### Nasal Vaccination

Specific pathogen-free 6- to 8-week-old female C57BL/6 (IgA^+/+^) mice were purchased from SLC Japan, and 6- to 8-week-old IgA-deficient (IgA^−/−^) mice (genetic C57BL/6 background) (17) were kindly provided by Dr. Tomoko Kurita-Ochiai from the Department of Microbiology and Immunology, Nihon University School of Dentistry at Matsudo and used in this study. Upon arrival, these mice were transferred to microisolators and maintained in horizontal laminar flow cabinets, and provided with sterile food and water as part of a specific pathogen-free facility at Osaka Dental University. Mice were immunized three times at weekly intervals nasally with 6 µl/nostril PBS containing 5 µg of *r*FimA plus 50 µg of pFL and 10 µg of CpG ODN as mucosal adjuvants (7–9). As controls, mice were immunized nasally with 5 µg of *r*FimA alone under the combination anesthesia with medetomidine, midazolam, and butorphanol. All experiments were conducted in accordance with the guidelines provided by Osaka Dental University. All of the mice used in these assays were free of bacterial and viral pathogens. This study conformed with the ARRIVE (Animal Research: Reporting of In Vivo Experiments) guideline for preclinical animal studies.

### Recombinant FimA (*r*FimA)

The DNA plasmid vector PYT1245 expressing whole FimA protein was kindly provided by Dr. Yutaka Terao at Niigata University (18). *Escherichia coli* BL21 competent cells (BioDynamics Laboratory Inc., Tokyo, Japan) were transformed with PYT1245 by the heat-shock method and were cultured in Luria-Bertani medium supplemented with ampicillin (100 µg/ml). The supernatants from ultrasonicated *E. coli* BL21 transformants carrying the PYT1245 plasmid were applied to a GST affinity column (Cytiva, Sheffield, UK). The *r*FimA protein was eluted by cleaving the GST-*r*FimA fusion protein with PreScission protease™ (Cytiva, Sheffield, UK). The recovered protein was applied to the affinity column (JNC CORPORATION, Tokyo, Japan) to remove endotoxin, and the elution was employed as the purified *r*FimA protein. The Limulus amebocyte lysate assay (BioWhittaker, Walkersville, MD, USA) resulted in <0.1 endotoxin units of LPS per 1 µg of *r*FimA.

### ELISA and ELISPOT Assays for *r*FimA-Specific Ab Responses

To assess *r*FimA-specific Ab levels, NWs, BALF, and plasma samples were collected 7 days after the last immunization and were then subjected to ELISA as described previously (19). Briefly, 96-well microtest assay plates (BD Biosciences, Oxnard, CA, USA) were coated with 1 µg/ml of *r*FimA in PBS. After incubating serial dilutions of samples, horseradish peroxidase-conjugated goat anti-mouse IgA or IgG Ab (Southern Biotechnology Associates Inc., Birmingham, AL, USA) were added to the wells. The color reaction was developed using 2,2’-azino-bis (3-ethylbenzothiazoline-6-sulphonic acid) (ABTS) substrate buffer for 15 min at room temperature. Endpoint titers were expressed as the reciprocal log_2_ of the last dilution that gave an OD_415_ of 0.1 greater than the background. Mice were euthanized 1 week after the final immunization by cervical spine fracture-dislocation under inhaled isoflurane anesthesia. Mononuclear cells were isolated from nasopharyngeal-associated lymphoid tissues (NALT), nasal passages (NPs), cervical lymph nodes (CLNs), lungs, mediastinal lymph nodes (MeLNs), and spleen and were then subjected to an enzyme-linked immunospot (ELISPOT) assay to enumerate the numbers of *r*FimA-specific IgA or IgG Ab-forming cells (AFCs) (19).

### Preparation of IgA-Enriched Samples

IgA-enriched samples were prepared by removing IgG and IgM Abs from NWs and BALF. NWs and BALF from mice immunized nasally with *r*FimA and FL/CpG or *r*FimA alone were applied on a protein G affinity mini-column (Protein G HP SpinTrap; Cytiva, Sheffield, UK), and the eluted solutions were further subjected to an IgM purification kit (Thermo Fisher Scientific, Tokyo, Japan). Approximately 800 µl of NWs and BALF were recovered by these procedures. These samples were serially diluted by PBS and subjected to live *P. gingivalis* cell-aggregation assays.

### Live *P. gingivalis* Cell-Aggregation Assay

*P. gingivalis* (500 µl; 3 × 10^8^ cells) in PBS were incubated with the serially-diluted IgA-enriched solutions (500 µl) from NWs or BALF in cuvettes. Five min later, the absorbance of the mixture was measured by a spectrophotometer (Eppendorf AG, Hamburg, Germany; OD 600 nm).

### Flow Cytometric Analysis

To characterize the phenotype of DCs, mononuclear cells were isolated from various mucosal tissues and lymph nodes 1 week after the last immunization with *r*FimA and FL/CpG or *r*FimA alone. The cells (0.2–1.0 × 10^6^ cells) were stained with Brilliant Violet 421-conjugated anti-mouse CD11c, and PE-labeled anti-mouse CD11b, CD8, or B220 mAbs (BioLegend, San Diego, CA, USA). In some experiments, mononuclear cells were incubated with Brilliant Violet 421-conjugated anti-mouse CD11c, and PE-labeled anti-mouse I-A^b^, CD40, CD80, or CD86 mAbs (BioLegend, San Diego, CA). These samples were then subjected to flow cytometry analysis (FACSVerse and Flow Jo; BD Biosciences, San Jose, CA, USA) (6).

### Cytokine Production by *r*FimA-Stimulated CD4^+^ T Cells

CD4^+^ T cells from NPs, CLNs, lungs, MeLNs, and spleens were purified by using an automatic cell sorter (AutoMACS^®^) system (Miltenyi Biotec B.V. & Co. KG, Bergisch Gladbach, Germany) as described previously (6, 20). The purified CD4^+^ T cell fraction (>97% CD4^+^ and >99% viable) was resuspended in RPMI 1640 (Sigma-Aldrich) supplemented with HEPES buffer (10 mM), L-glutamine (2 mM), nonessential amino acid solution (10 µl/ml), sodium pyruvate (10 mM), penicillin (100 U/ml), streptomycin (100 µg/ml), gentamycin (80 µg/ml), and 10% FCS (complete medium; 4 × 10^6^ cells/ml), and cultured in the presence of T cell-depleted, complement-, and mitomycin-treated splenic Ag-presenting cells taken from naïve BALB/c mice with or without *r*FimA (2 µg/ml). The culture supernatants were collected on day five and analyzed by using IFN-γ-, IL-2-, IL-4-, IL-5-, and IL-6-specific ELISA kits (Invitrogen, Carlsbad, CA, USA). The detection limits for each cytokine were: 5.3 pg/ml for IFN-γ, 2.0 pg/ml for IL-2, 4 pg/ml for IL-4, 3.3 pg/ml for IL-5, and 6.5 pg/ml for IL-6.

### *P. gingivalis* Clearance in the Upper and Lower Respiratory Tracts

One week after the last immunization, IgA^+/+^ and IgA^−/−^ (genetic background C57BL/6) mice were nasally challenged with the *P. gingivalis* 381 strain at a dose of 1 × 10^8^ cfu (100 µl). Two days after the bacterial challenge, NWs were harvested aseptically by flushing with 1 ml of sterile PBS from the choana. After the lungs were removed with the tracheae, BALF was collected by gentle injection of 1 ml sterile PBS into the lungs from the tracheae. One hundred µl of NWs and BALF were spread on agar medium including kanamycin and were cultivated for 7 days at 37°C under anaerobic conditions.

### Statistical Analysis

The data are expressed as the mean ± standard error of the mean (SEM). All mouse groups were compared to control mice with an unpaired Mann-Whitney U test using GraphPad Prism version 7 (GraphPad Software, Inc., La Jolla, CA, USA). *p* values of <0.05 were considered statistically significant.

## Results

### Induction of *r*FimA-Specific Ab Responses in Mucosal and Systemic Tissues With FL/CpG Adjuvant

**Figure 1:**
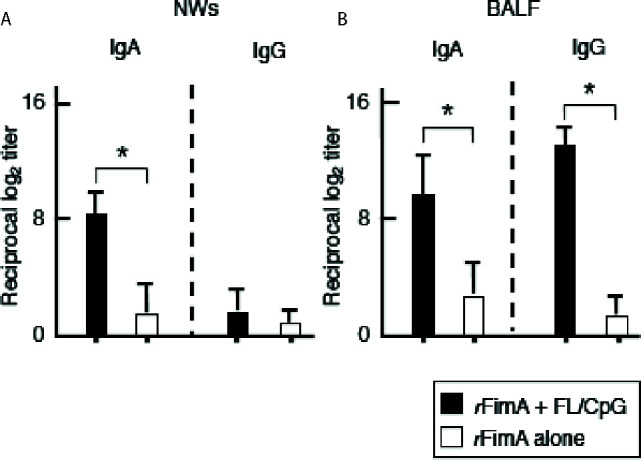
*P. gingivalis r*FimA-specific Ab responses in the external secretions. C57BL/6 (6–8 weeks) mice were nasally immunized three times at weekly intervals with *r*FimA (10 µg) plus pFL (50 µg) and CpG ODN (10 µg) (filled bars), or *r*FimA (10 µg) alone (open bars). Seven days after the last immunization, the levels of *r*FimA-specific IgA and IgG Abs in NWs **(A)** and BALF **(B)**, were determined by *r*FimA-specific ELISA. The values shown are the mean ± SE (n = 20). *p < 0.05 when compared with mice given *r*FimA alone.

**Figure 2:**
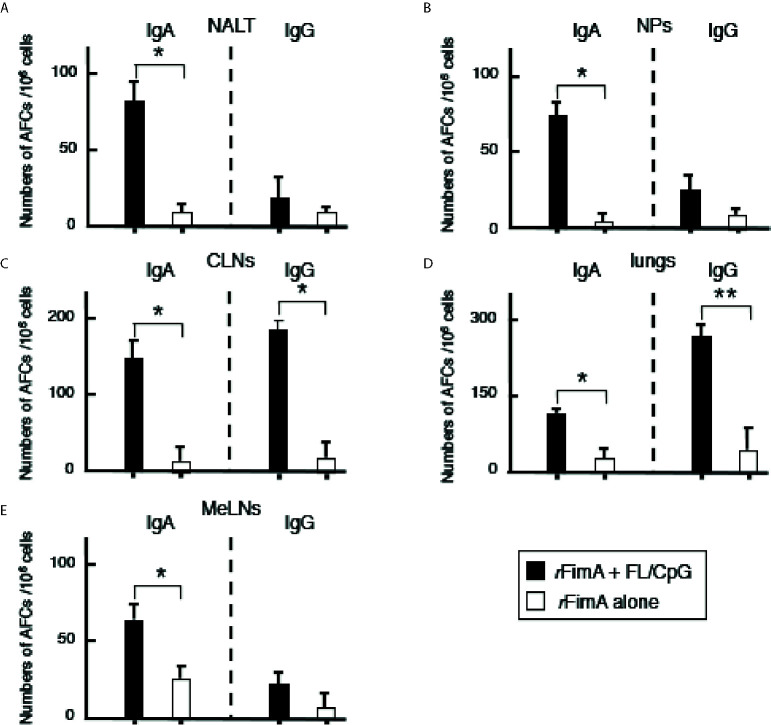
Antibody forming cells in the respiratory tracts, Mice were nasally immunized as described in **Figure 1** legend. Seven days after the last immunization, mononuclear cells were isolated from NALT **(A)**, NPs **(B)**, CLNs **(C)**, lungs **(D)**, and MeLNs **(E)**, and were then subjected to ELISPOT assays to enumerate the numbers of Ag-specific IgG and IgA AFCs. The values shown are the mean ± SE (n = 20). *p < 0.05 and **p < 0.01 when compared with mice given *r*FimA alone.

**Figure 3:**
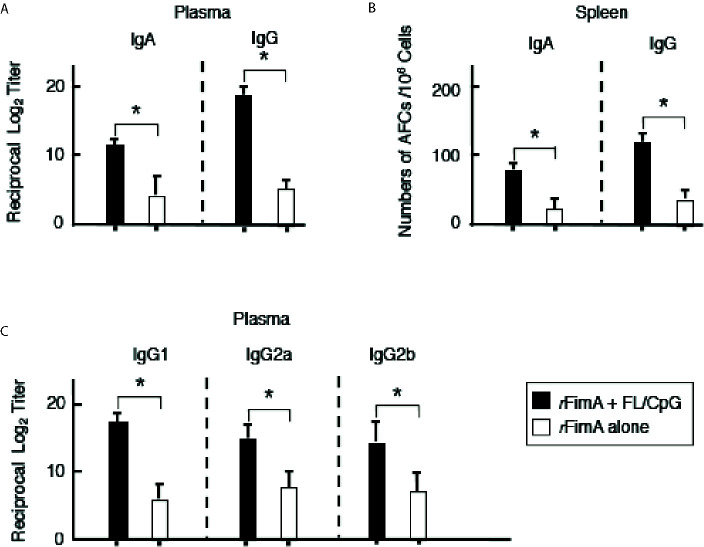
*P. gingivalis r*FimA-specific Ab responses in plasma and spleens. Mice were nasally immunized as described in **Figure 1** legend. Seven days after the last immunization, the levels of *r*FimA-specific IgA, IgG, and IgG subclass Abs in plasma **(A, C)** were determined by *r*FimA-specific ELISA. In some experiments, mononuclear cells were isolated from spleen **(B)**, and were then subjected to ELISPOT assays to enumerate the numbers of Ag-specific IgG and IgA AFCs. The values shown are the mean ± SE (n = 20). *p < 0.05 when compared with mice given *r*FimA alone.

We initially examined whether nasal administration of FL/CpG as a mucosal adjuvant would enhance *r*FimA-specific Ab responses. Mice given nasal *r*FimA plus FL/CpG had significantly increased levels of *r*FimA-specific IgA Ab responses in NWs when compared with mice given nasal *r*FimA alone (**Figure 1A**). Further, significantly elevated levels of *r*FimA-specific IgA and IgG Abs were seen in BALF of mice given nasal *r*FimA plus FL/CpG when compared with Ab levels in the controls (**Figure 1B**). These findings were further confirmed at the cellular level by using enzyme-linked immunospot (ELISPOT) assays. Elevated numbers of *r*FimA-specific IgA antibody-forming cells (AFCs) were detected in the mucosal inductive and effector tissues as well as their draining lymph nodes of mice given nasal *r*FimA plus FL/CpG (**Figures 2A–E**). In addition, increased numbers of anti-*r*FimA-specific IgG AFCs were seen in cervical lymph nodes (CLNs) and lungs (**Figures 2C, D** **)**. Since nasal immunization is known to induce systemic immune responses in addition to mucosal immunity, *r*FimA-specific Ab responses in plasma and spleen were examined. Nasal administration with FL/CpG as a mucosal adjuvant successfully enhanced *r*FimA-specific IgG and IgA Ab responses in plasma (**Figure 3A**). Thus, significantly higher numbers of *r*FimA-specific IgG and IgA AFCs were also seen in spleens of mice given FL/CpG than in mice given *r*FimA alone (**Figure 3B**). Of importance, 6 months after the last immunization with *r*FimA plus FL/CpG, IgA Ab responses in NWs were maintained (Reciprocal Log_2_ titer 7.2). Further, *r*FimA-specific IgA and IgG Ab responses in BALF as well as those in plasma are essentially the same as those responses detected at 4 weeks after the initial immunization. When *r*FimA-specific IgG subclass Ab levels were examined, increased anti-*r*FimA IgG1, IgG2a, and IgG2b Abs were noted in mice given nasal *r*FimA plus FL/CpG when compared with those Ab responses in mice given *r*FimA alone (**Figure 3C**). Essentially no IgG3 Ab response against *r*FimA was detected (data not shown). Taken together, these findings show that the nasal FL/CpG system effectively upregulated *r*FimA-specific Ab responses in both mucosal and systemic immune compartments.

### Nasal *r*FimA Plus pFL and CpG ODN Induce CD11b^+^ and CD8^+^ DCs in the Respiratory Tract

Since our previous studies reported that nasal administration with FL/CpG as a mucosal adjuvant resulted in increased numbers of matured-type CD11c^+^ DCs, which contribute to the induction of specific immune responses to ovalbumin (7), recombinant pneumococcal surface protein A (PspA) (8), or hemagglutinin of influenza virus (9), we next characterized CD11c^+^ DCs in the various mucosal tissues of mice given *r*FimA plus FL/CpG or *r*FimA alone. Nasal immunization of *r*FimA plus FL/CpG significantly increased the frequency of CD11c^+^ cells in the NALT, NPs, MeLNs, lungs, and CLNs when compared with mice given *r*FimA alone (**Table 1**, **Supplementary Figure 1**). The numbers of CD8^+^- and CD11b^+^-DC subsets were significantly increased in mucosal tissues of mice given FL/CpG as a nasal adjuvant when compared with mice given nasal *r*FimA alone (**Table 1**). Of interest, the expanded CD11c^+^ DCs in NALT, NPs, MeLNs, lungs, and CLNs expressed higher levels of MHC II, CD40, CD80, and CD86 molecules (**Table 1**). Taken together, these results indicate that nasal vaccination with *r*FimA plus FL/CpG preferentially expands the numbers of matured-type CD8^+^- and CD11b^+^-DC populations, which are most likely involved in the induction of *r*FimA-specific T and B cell responses.

**Table 1:** Frequencies of CD11c^+^ DCs and CD8, CD11b, and B220, and costimulatory molecule expressions by CD11c^+^ DCs in mucosal inductive and effector tissues of mice given nasal *r*FimA with or without FL/CpG*^b^*.

Tissue	FL/CpG	%of CD11c^+^ cells/total lymphocytes	% of total CD11c^+^ DCs						
			CD8*^c^*	CD11b*^c^*	B220*^c^*	CD40*^c^*	CD80*^c^*	CD86*^c^*	MHC II*^c^*
NALT	+	*6.7 ± 1.1	*20.1 ± 2.0	*33.4 ± 4.8	48.1 ± 5.6	*5.1 ± 0.9	*9.9 ± 2.4	*15.2 ± 3.1	*81.1 ± 8.8
	−	1.9 ± 0.3	11.1 ± 1.6	14.1 ± 3.9	47.5 ± 4.7	0.8 ± 0.2	5.6 ± 1.8	6.9 ± 2.2	44.2 ± 9.3
NPs	+	*12.3 ± 2.8	*28.2 ± 2.4	*39.7 ± 5.5	21.4 ± 2.8	*8.6 ± 2.9	15.4 ± 4.2	23.3 ± 8.8	*45.5 ± 8.4
	−	4.9 ± 1.8	13.7 ± 2.7	25.9 ± 3.1	19.9 ± 2.5	3.9 ± 1.3	16.7 ± 3.4	22.4 ± 5.7	35.1 ± 5.8
MeLNs	+	*5.4 ± 1.3	*22.4 ± 3.5	*28.8 ± 3.3	39.5 ± 6.2	4.6 ± 1.2	*16.6 ± 4.1	*15.5 ± 4.5	*75.8 ± 14
	−	1.7 ± 0.5	8.5 ± 2.8	14.1 ± 2.8	36.6 ± 5.6	1.2 ± 0.5	6.6 ± 2.4	7.1 ± 2.6	31.7 ± 5.6
Lungs	+	*12.4 ± 2.6	*23.4 ± 5.1	*30.3 ± 6.7	22.5 ± 3.4	7.2 ± 1.9	*18.8 ± 3.6	*19.9 ± 5.2	*68.2 ± 9.7
	−	2.8 ± 0.5	9.7 ± 2.6	14.4 ± 3.8	19.9 ± 4.2	3.2 ± 2.1	7.7 ± 3.8	6.8 ± 3.5	30.5 ± 8.7
CLNs	+	*2.9 ± 0.4	*28.7 ± 6.1	*31.1 ± 8.5	38.1 ± 8.8	2.8 ± 0.6	*12.2 ± 3.4	*48.6 ± 6.2	*88.2 ± 11
	−	1.3 ± 0.4	15.3 ± 2.6	23.5 ± 8.6	33.6 ± 6.6	0.9 ± 0.4	7.9 ± 2.9	29.3 ± 5.5	77.4 ± 12
Spleen	+	*4.5 ± 1.1	*15.2 ± 4.2	27.4 ± 6.3	33.8 ± 9.5	8.4 ± 3.2	14.7 ± 4.0	24.4 ± 8.9	40.8 ± 8.8
	−	2.2 ± 0.7	7.1 ± 2.6	18.7 ± 4.7	30.1 ± 9.8	7.3 ± 3.1	13.4 ± 5.1	25.8 ± 7.7	36.3 ± 5.2

Mice were nasally immunized weekly for 3 consecutive weeks with 10 µg of rFimA as Ag plus 50 µg of pFL and 10 µg of CpG ODN, or 10 µg of rFimA alone. One week after the final administration, mononuclear cells from NALT, NPs, MeLNs, lungs, CLNs, and Spleen were stained with a combination of the respective mAbs and subjected to flow cytometry analysis by FACSVerse.

^a^Mononuclear cells were stained with Brilliant Violet 421-labeled anti-mouse CD11c mAb.

^b^Values shown are means ± SEM of five independent experiments. Each group consists of five mice.

^c^Mononuclear cells were stained with Brilliant Violet 421-labeled anti-mouse CD11c and PE-tagged anti-mouse CD8, anti-mouse 11b, anti-mouse B220, anti-mouse CD40, anti-mouse CD80, anti-mouse CD86, or anti-mouse I-A^d^.

*p < 0.05 when compared with mice immunized with rFimA alone.

### Th1- and Th2-Type Cytokine Responses by *r*FimA-Stimulated Mucosal CD4^+^ T Cells

We next assessed cytokines production by *r*FimA-stimulated CD4^+^ T cells in NPs, MeLNs, lungs, CLNs, and spleens of mice given nasal *r*FimA plus FL/CpG or *r*FimA alone. *r*FimA-stimulated CD4^+^ T cells from NPs and lungs of mice given FL/CpG as a nasal adjuvant exhibited significantly higher levels of IFN-γ, IL-2, IL-4, and IL-5 production than in control mice, though the levels of IL-6 synthesis were unchanged (**Table 2**). *r*FimA-stimulated CD4^+^ T cells from MeLNs and CLNs of mice given *r*FimA plus FL/CpG displayed significantly higher levels of IFN-γ, IL-2, and IL-4 production. In addition, splenic CD4^+^ T cells isolated from mice given nasal FL/CpG showed increased levels of Th1- and Th2-type cytokine responses when compared with those in mice given *r*FimA alone. These results show that FL/CpG as a nasal adjuvant provokes a balanced Th1- and Th2-type cytokine response in the lower and upper respiratory mucosa as well as in the spleens.

**Table 2:** Th1- and Th2-type cytokine responses by CD4^+^ T cells after *in vitro* restimulation with *r*FimA.

Tissue	FL/CpG	Production of Th1- or Th2-type Cytokines				
		IFN-γ (ng/ml)	IL-2 (pg/ml)	IL-4 (pg/ml)	IL-5 (pg/ml)	IL-6 (pg/ml)
NPs	+	*1.4 ± 0.3	**18 ± 4.7	**32 ± 5.2	**29 ± 11	201 ± 32
	−	0.8 ± 0.2	5.5 ± 1.9	11 ± 3.8	12 ± 3.8	127 ± 41
MeLNs	+	**2.8 ± 1.1	**32 ± 12	**42 ± 6.8	21 ± 7.0	356 ± 48
	−	0.9 ± 0.5	6.4 ± 2.5	9.1 ± 4.1	15 ± 5.3	264 ± 33
Lungs	+	*2.3 ± 1.0	**44 ± 10	**55 ± 12	*42 ± 14	400 ± 46
	−	1.5 ± 0.6	6.6 ± 2.1	13 ± 5.0	29 ± 8.7	232 ± 64
CLNs	+	**3.3 ± 1.1	**35 ± 7.2	**22 ± 7.1	12 ± 4.2	160 ± 71
	−	0.5 ± 0.2	6.1 ± 2.1	2.5 ± 0.8	8.8 ± 2.4	149 ± 11
Spleen	+	*1.7 ± 0.3	*11.5 ± 3.7	*24 ± 5.9	23 ± 4.4	401 ± 46
	−	0.7 ± 0.3	5.0 ± 2.1	9.7 ± 3.1	12 ± 4.9	288 ± 30

Each group of mice was nasally immunized weekly for 3 consecutive weeks with 10 µg of rFimA plus 50 µg of pFL and 10 µg of CpG ODN, or 10 µg of rFimA alone. One week after the final administration, CD4^+^ T cells (4 × 10^6^ cells/ml) from NPs, MeLNs, lungs, CLNs, and Spleens were cultured with 500 µg/ml of rFimA in the presence or absence of T cell-depleted splenic feeder cells (8 × 10^6^ cells/ml).

^a^Culture supernatants were harvested after 5 days of incubation and analyzed by the respective cytokine-specific ELISA. The levels of each cytokine are expressed by subtracting the protein value of non-stimulated cultures from stimulated cultures.

Values shown are means ± SEM of three independent experiments. Each group consists of five C57BL/6 mice.

*p < 0.05, **p < 0.01 when compared with mice immunized with rFimA alone.

### Interactions Between NWs/BALF SIgA Abs and Live *P. gingivalis* Cells

**Figure 4:**
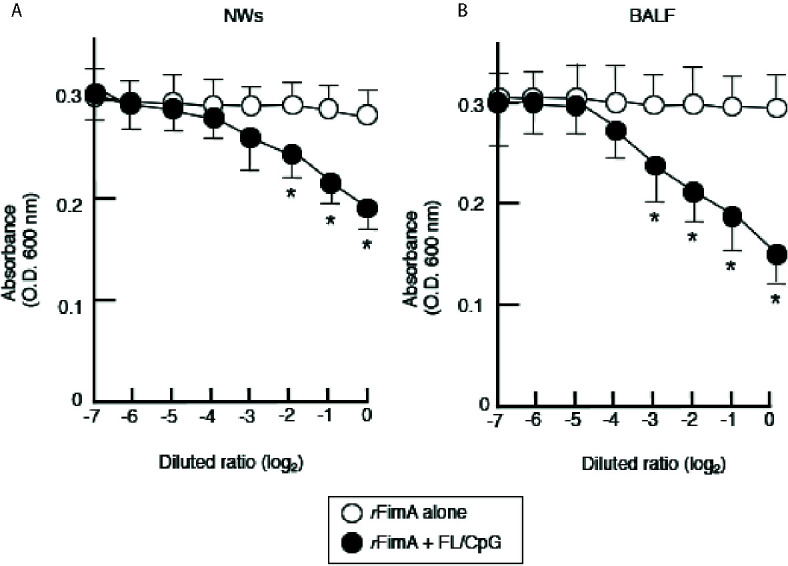
Aggregation of live *P. gingivalis* cells by SIgA Abs. Enriched SIgA Abs from NWs **(A)** and BALF **(B)** of mice given nasal *r*FimA plus FL/CpG (filled circles) or *r*FimA alone (open circles) were serially diluted with PBS. The serial-diluted IgA-enriched solutions (500 µl) from NWs or BALF were added to 3 × 10^8^ *P. gingivalis* cells (500 µl) in cuvettes for 5 min. The optical density of the mixture was measured by a spectrophotometer (OD 600 nm). The values shown are the means ± SE (n = 5). *p < 0.05, when compared with mice given *r*FimA alone.

Thus far, our results showed that nasal FL/CpG as a mucosal adjuvant effectively activated mucosal DCs for the induction of Th1- and Th2-type cytokine-mediated *r*FimA-specific Ab responses. We next assessed the functional property of *r*FimA-specific IgA Abs by the aggregation of live *P. gingivalis* cells. SIgA Abs in NWs and BALF were enriched by removing IgG and IgM Abs using respective affinity columns. When live *P. gingivalis* (1 × 10^8^) were incubated with enriched SIgA Abs from NWs and BALF of mice given FL/CpG as a nasal adjuvant, a significant aggregation of *P. gingivalis* was induced in the Ab concentration-dependent manner (**Figures 4A, B** **)**. In contrast, the enriched SIgA Ab from mice given Ag alone exhibited essentially no live *P. gingivalis* cell aggregation (**Figures 4A, B** **)**. We also performed that the *P. gingivalis* aggregation assay using unpurified NW and BALF. The levels of the aggregation are essentially the same when purified NW and BALF were employed (data not shown). These results indicate that *r*FimA-specific SIgA SIgA, but not IgG nor IgM Abs in NW and BLAF play a key role in the aggregation of *P. gingivalis.*

### An Essential Role for SIgA Abs in the Protection Against Respiratory *P. gingivalis* Infection

**Figure 5:**
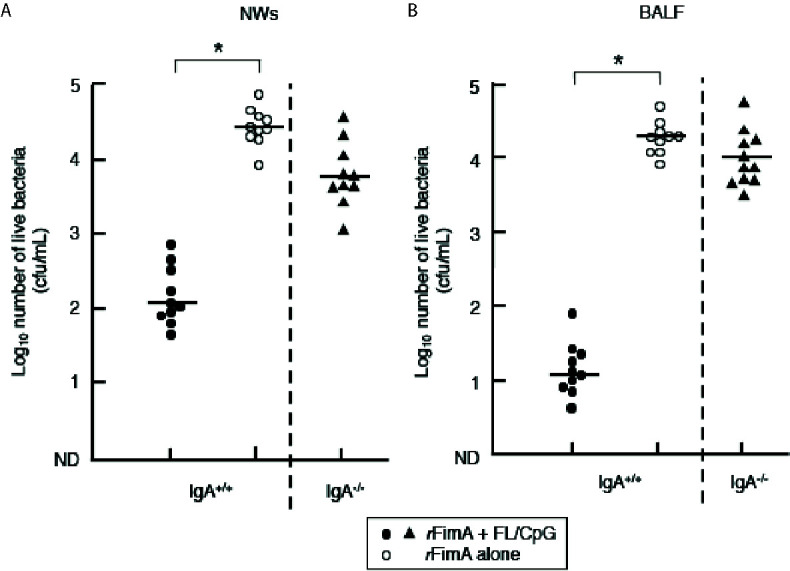
Clearance of *P. gingivalis* cells in the upper and lower respiratory tracts. IgA^+/+^ and IgA^−/−^ mice given nasal FL/CpG ODN-based *r*FimA vaccine (filled circles or triangles). As a control, mice were nasally immunized with *r*FimA alone (open circles). One week after the last immunization, all groups of mice were nasally challenged with *P. gingivalis* cells (1 × 10^8^ cfu). Two days after the bacterial challenge, NWs **(A)** and BALF **(B)** samples were collected. The samples (100 µl) were cultured anaerobically on kanamycin-supplemented blood agar plates to enumerate the recovered CFU. The values shown are the means ± SE (n = 10). Each line represents the median log_10_ CFU/mouse. *p < 0.05 when compared with mice given *r*FimA alone.

To further determine the essential roles of *r*FimA-specific SIgA Abs induced by nasal vaccination with *r*FImA plus FL/CpG ODN, both IgA-deficient (IgA^−/−^) and the genetic background control (IgA^+/+^) C57BL/6 mice were nasally challenged with the *P. gingivalis* 381 strain (0.2 × 10^8^ cfu/20 µl per shot, 1 × 10^8^ cfu consecutive inoculation of a total of five shots) 1 week after the last immunization. Two days after the bacterial challenge, NWs and BALF samples were collected, and the samples were cultured anaerobically on kanamycin-supplemented blood agar plates to enumerate the recovered CFU. IgA^+/+^ mice given nasal *r*FimA plus FL/CpG showed significantly low numbers of bacterial CFUs (**Figure 5**). Conversely, NWs and BALF of IgA^+/+^ mice given *r*FimA alone contained high numbers of *P. gingivalis* (**Figure 5**, **Supplementary Figure 2**). These results show that nasal *r*FimA plus FL/CpG provides effective protection against *P. gingivalis* colonization in the lungs and nasal cavity. Of importance, when IgA^−/−^ mice given *r*FimA plus FL/CpG ODN were challenged nasally with the *P. gingivalis* 381 strain, high numbers of bacterial colonies were noted in NWs and BALF, which were comparable to those seen in NWs and BALF of IgA^+/+^ mice given *r*FimA alone (**Figure 5**).

## Discussion

In this study, we investigated whether nasal pFL and CpG ODN (FL/CpG) as a mucosal adjuvant could elicit bacterial Ag (*r*FimA)-specific Ab responses to protect against *P. gingivalis* respiratory infection. Our results showed that the nasal FL/CpG adjuvant system successfully provoked *r*FimA-specific mucosal and systemic immune responses through the expansion of CD8- and CD11b-expressing DC populations and the induction of Th1- and Th2-type cytokine responses by CD4^+^ T cells. In addition, anti-*r*FimA SIgA Abs induced by nasal FL/CpG effectively purged *P. gingivalis* in the lower and upper respiratory mucosa. It is well known that pathogen-specific SIgA Ab plays key roles in the protection and homeostatic regulation of mucosal epithelia, including the oral cavity and the respiratory tracts, of humans and many other mammals (21). Indeed, our previous study showed that pFL as a nasal adjuvant elicits pneumococcal surface protein A (PspA)-specific SIgA Ab responses in the nasal cavity, which prevents nasal carriage of *S. pneumoniae* (22). In addition, it has been shown that hemagglutinin-specific SIgA Abs are essential for the prevention of influenza virus infection in the nasal mucosa (9). These results indicate that Ag-specific SIgA Abs are required for protection against the initial step of bacterial and viral infection, which mainly occurs in the nasal mucosa. To this end, previous studies have shown that the clearance of bacterial or viral infections in the lower respiratory tract can be achieved without pathogen-specific SIgA Ab responses (23, 24). According to these findings, our current study is the first to show that *P. gingivalis*-specific functional SIgA Abs play an indispensable role in the clearance of oral pathogenic bacteria in the lower respiratory tract in addition to the nasal cavity.

The induction of Ag-specific IgG Ab is essential for the prevention of systemic infection. In this study, increased levels of *r*FimA-specific IgG Ab responses were detected in the respiratory tract. Unfortunately, these *r*FimA-specific IgG Abs did not show the protective activity, since IgA^−/−^ mice given nasal FL/CpG vaccine failed to eliminate *P. gingivalis* from the respiratory tract. However, we anticipated that *r*FimA-specific IgG Abs could play essential roles when *P. gingivalis* invades into the systemic circulation of the host. Indeed, FL/CpG as combined mucosal adjuvant elicited a balanced Th1- and Th2-type cytokine responses, we predict that *r*FimA-specific IgG1, IgG2a, and IgG2b Ab responses were induced in plasma. Thus, it is possible that these IgG Abs could participate in the opsonization and/or antibody-dependent cellular cytotoxicity (ADCC) activities.

Since populations are aging all over the world, including in Japan, it is important to maintain healthy conditions in the elderly. However, it has been reported that the number of pneumonia patients is increasing in Japan in step with the growing aged population due to poor oral hygiene or a decrease in salivary clearance (25). Furthermore, it has been reported that 5–15% of cases of pneumonia in the hospitalized population are aspiration pneumonia (26). In this regard, many people aged 65 or older die of pneumonia, including aspiration pneumonia (27). Since some pathogenic bacteria causing aspiration pneumonia are anaerobes, including periodontal pathogens such as *P. gingivalis* that originate in the oral cavity (11, 28), antimicrobial agents targeting anaerobic bacteria are prescribed to aspiration pneumonia patients in order to control and treat inflammation and infection. For prophylaxis of aspiration pneumonia, oral cleaning and oral healthcare of the elderly can promote quality of life or prolong life expectancy (29), since good oral hygiene leads to the prevention of aspiration pneumonia (30). Based upon these reports, it is possible that the effective inhibition of oral, nasopharyngeal, and pulmonary bacterial colonization may lead to a drastic reduction of bacterial growth and subsequently prevent aspiration pneumonia by *P. gingivalis*.

Our current study clearly showed that a nasal vaccine consisting of *r*FimA and FL/CpG elicited functional *r*FimA-specific SIgA Ab responses in the nasal cavity and lungs, and thus vaccinated mice exhibited complete protection in the lower and upper respiratory tracts when nasally challenged with a large amount (1.0 × 10^8^ cfu) of *P. gingivalis* strain 381. In contrast, mice given nasal *r*FimA alone showed significantly increased numbers of bacteria in the BALF and NWs after being challenged nasally with *P. gingivalis* compared to mice given *r*FimA and FL/CpG. In addition, we showed that this nasal vaccination strategy induced *r*FimA-specific IgA Ab responses in the saliva (3), that blocked *P. gingivalis* binding to a salivary protein (statherin) on the hydroxyapatite beads. Therefore, *r*FimA-specific salivary IgA could reduce the number of *P. gingivalis* in the oral cavity that contributes to the prevention of aspiration pneumonia by *r*FimA-specific IgA Ab in the respiratory tracts. Furthermore, the nasal FL/CpG system has been shown to elicit pathogen-specific SIgA Ab responses in aged mice (3, 8, 9). To this end, one can predict that nasal *r*FimA plus FL/CpG is a potent strategy to prevent aspiration pneumonia not only in young adults but also in aged patients with periodontitis. Therefore, we are currently testing whether the nasal vaccine with *r*FimA and FL/CpG can induce protective *r*FimA-specific SIgA Ab responses in aged mice.

A combination of pFL and CpG ODN has been employed as a DC-targeting nasal adjuvant for the induction of functional CD4^+^ T cells and pathogen-specific mucosal immunity (8, 9, 31). Thus, increased numbers of mature-type CD8- or CD11b-expressing DC subsets that co-express MHC II, CD40, CD80, and CD86 molecules have been noted in NALT, NPs, and CLNs (8, 9). Furthermore, others have also reported that the induction of CD8^+^ or CD11b^+^ DC subsets is essential for protection against respiratory *Bordetella pertussis* infection (32) or influenza virus infection (33). The current study clearly agrees with these previous findings. Thus, a nasal vaccine composed of FL/CpG and *r*FimA significantly elicited mature-type CD8^+^ or CD11b^+^ DCs for the subsequent induction of *P. gingivalis*-specific protective immunity in the respiratory tract. Our previous study showed that pFL as nasal adjuvant resulted in CD8^+^ DCs-mediated Th2-type cytokine responses. In the present study, when CpG was added to pFL as a nasal adjuvant, both Th1-and Th2-type cytokine responses and increased number of CD8^+^ DCs and CD11b^+^ DCs were noted. Thus, it is possible that nasal CpG is known as Th1-mediated nasal adjuvant (34) preferentially elicit CD11b^+^ DCs. To support this view, nasal adenovirus expressing FL induced increased numbers of CD11b^+^ DCs with humoral and cellular immunity (35). Further, this study showed that the migration of DCs from NALT to CLNs, NP, and SMGs (35). Thus, increased numbers of certain subsets of DCs in the various lymphoid tissues of mice given nasal *r*FimA plus FL/CpG could be attributed to DC migration from NALT to these tissues. Similarly, CD11b^+^ DCs in the lamina propria of the small intestine contribute both humoral and cellular immunity (36), while CD103 expressing CD8^+^ DCs in the lamina propria are prone to induce Th1-type responses with CTL activity (37). Taken together, a vaccine containing FL/CpG as nasal adjuvant potentially induce CD8^+^ and CD11b^+^ DCs for facilitating the induction of Th1- and Th2-type cytokine-mediated humoral and cellular immune responses. Since it has been reported that interactions between DCs and CD4^+^ T cells play an important role in the induction of pulmonary immunity (38), we next investigated Th1- and Th2-type cytokine responses by *r*FimA-stimulated CD4^+^ T cells in various mucosal tissues of mice given nasal *r*FimA and FL/CpG. *r*FimA-stimulated CD4^+^ T cells from NPs, lungs, MeLNs, and CLNs of mice given *r*FimA and FL/CpG exhibited significantly higher levels of Th1- and Th2-type cytokine production when compared with those in control mice (**Table 2**). These results also agree with our previous studies that showed that a balanced Th1- and Th2-type cytokine response occurred in mice given nasal FL/CpG as a mucosal adjuvant (7–9). It has been shown that both Th1- and Th2-type responses contribute to the induction of Ag-specific mucosal IgA Ab responses (39, 40). However, it has been also suggested that polarized either Th1- or Th2-type response could elicit undesired inflammation or allergy (41). Thus, a balanced Th1- and Th2-type response by DC-targeting pFL/CpG nasal adjuvant is essential for the development of safe vaccines. Taken together, the nasal FL/CpG system with *r*FimA as Ag provoked mucosal and systemic immunity, which were mediated by the upregulation of CD8- and CD11b-expressing DC populations and Th1-/Th2-type cytokine-producing CD4^+^ T cells.

In summary, our present study clearly showed that nasal *r*FimA and FL/CpG as a mucosal adjuvant provoked CD8- or CD11b-expressing DCs and Th1- and Th2-type cytokine-producing CD4^+^ T cells for the induction anti-*r*FimA SIgA Abs, which play an indispensable role in protection against *P. gingivalis* infection in the respiratory mucosa. These findings are the first to show that nasal vaccine-induced bacterial Ag (FimA)-specific SIgA Abs are potent for the prevention of *P. gingivalis*-mediated aspiration pneumonia.

## Data Availability Statement

The raw data supporting the conclusions of this article will be made available by the authors, without undue reservation.

## Ethics Statement

The animal study was reviewed and approved by Osaka Dental University animal experimental committee.

## Author Contributions

KKa and KF contributed to the conception, design, data acquisition, analysis, and interpretation of the study, and drafted and critically revised the manuscript. SK contributed to the conception, design, data acquisition, analysis, and interpretation of the study, and critically revised the manuscript. YH contributed to the study conception and critically revised the manuscript. KKo and TM contributed to the data acquisition and analysis, and critically revised the manuscript. All authors contributed to the article and approved the submitted version.

## Funding

This work is supported by the Japan Society for the Promotion of Science (JEPS) KAKENHI Grant Numbers JP17H04424 (B) and JP17K12034 (C) to KKa, as well as JP20H03856 (B) and JP20K20495 to KF from the Ministry of Education, Science, Sports, and Culture of Japan. KKa is a recipient of a grant from the Central R&D Laboratory, Kobayashi Pharmaceutical Co., Ltd.

## Conflict of Interest

The authors declare that the research was conducted in the absence of any commercial and financial relationships that could be construed as a potential conflict of interest.

The reviewer SM declared a shared affiliation with one of the authors, KF, to the handling editor at the time of review.

The reviewer ZM declared a shared affiliation with one of the authors, KF, to the handling editor at the time of review.

## Supplementary Material

Supplementary Figure 1 Typical FACS plot and gating strategy for NALT and lungs. Mononuclear cells in NALT and lungs were gated by using the forward- and side-scatter properties, and were subsequently analyzed for CD11c^+^ cells.

Supplementary Figure 2 Agar medium plates incubated with NWs or BALF of IgA^+/+^ mice given nasal *r*FimA and FL/CpG or *r*FimA alone and IgA^−/−^ mice given nasal *r*FimA and FL/CpG. One hundred µl of NWs and BALF were spread on agar medium including kanamycin and were cultivated for 144 h at 37°C under anaerobic conditions.

## Abbreviations

FL, Flt3 ligand; pFL, a DNA plasmid encoding; CpG ODN, CpG oligodeoxynucleotide; FL/CpG, pFL and CpG ODN; Ab, antibody; Ag, antigen; *r*FimA, recombinant FimA; NWs, Nasal Washes; BALF, bronchoalveolar lavage fluid; SIgA, secretory IgA; DCs, dendritic cells; NALT, nasopharyngeal-associated lymphoid tissues; NPs, nasal passages; CLNs, cervical lymph nodes; MLNs, mediastinal lymph nodes; *P. gingivalis*, *Porphyromonas gingivalis*.
