# Supplementary figures and images for: Respiratory FimA-Specific Secretory IgA Antibodies Upregulated by DC-Targeting Nasal Double DNA Adjuvant Are Essential for Elimination of Porphyromonas gingivalis
Source: Front Immunol. 2021 Feb 25;12:634923. doi: 10.3389/fimmu.2021.634923 (PMC7948520; doi:10.3389/fimmu.2021.634923)

## Supplementary Figure 1

NALT

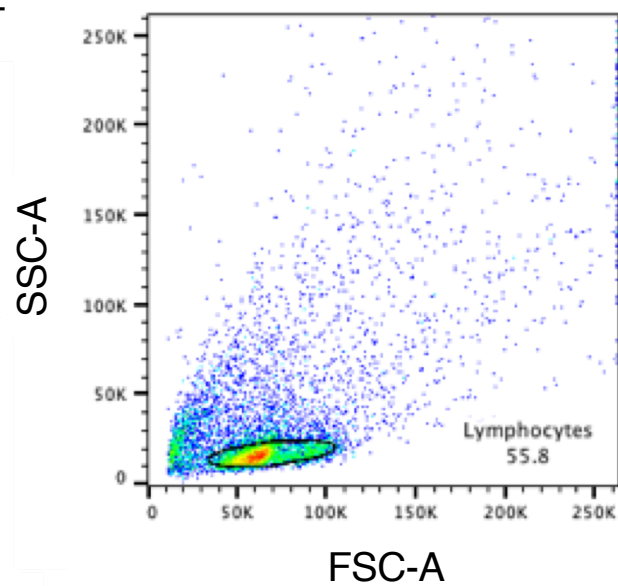

Count

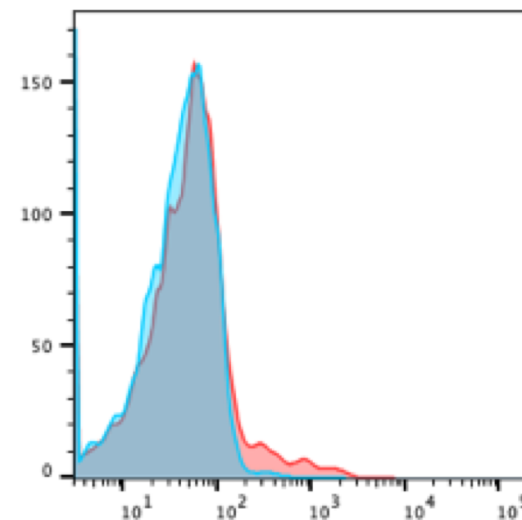

lungs

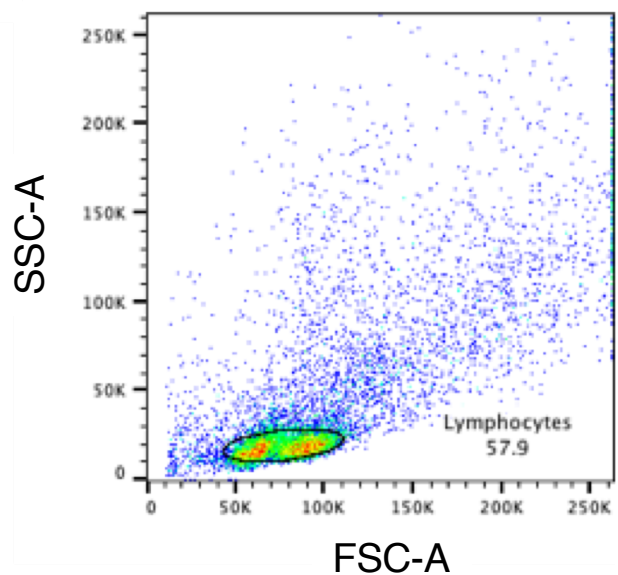

Count

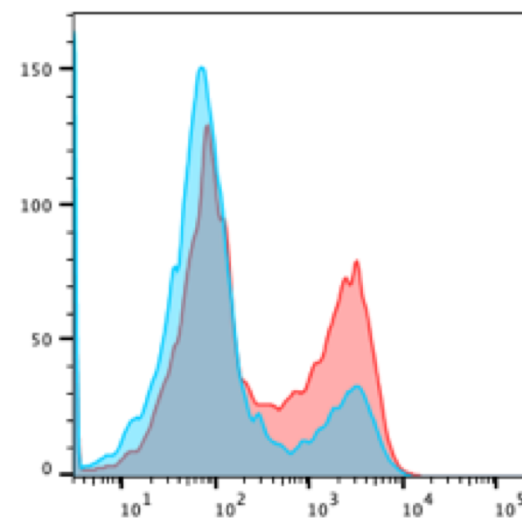

## Supplementary Figure 2

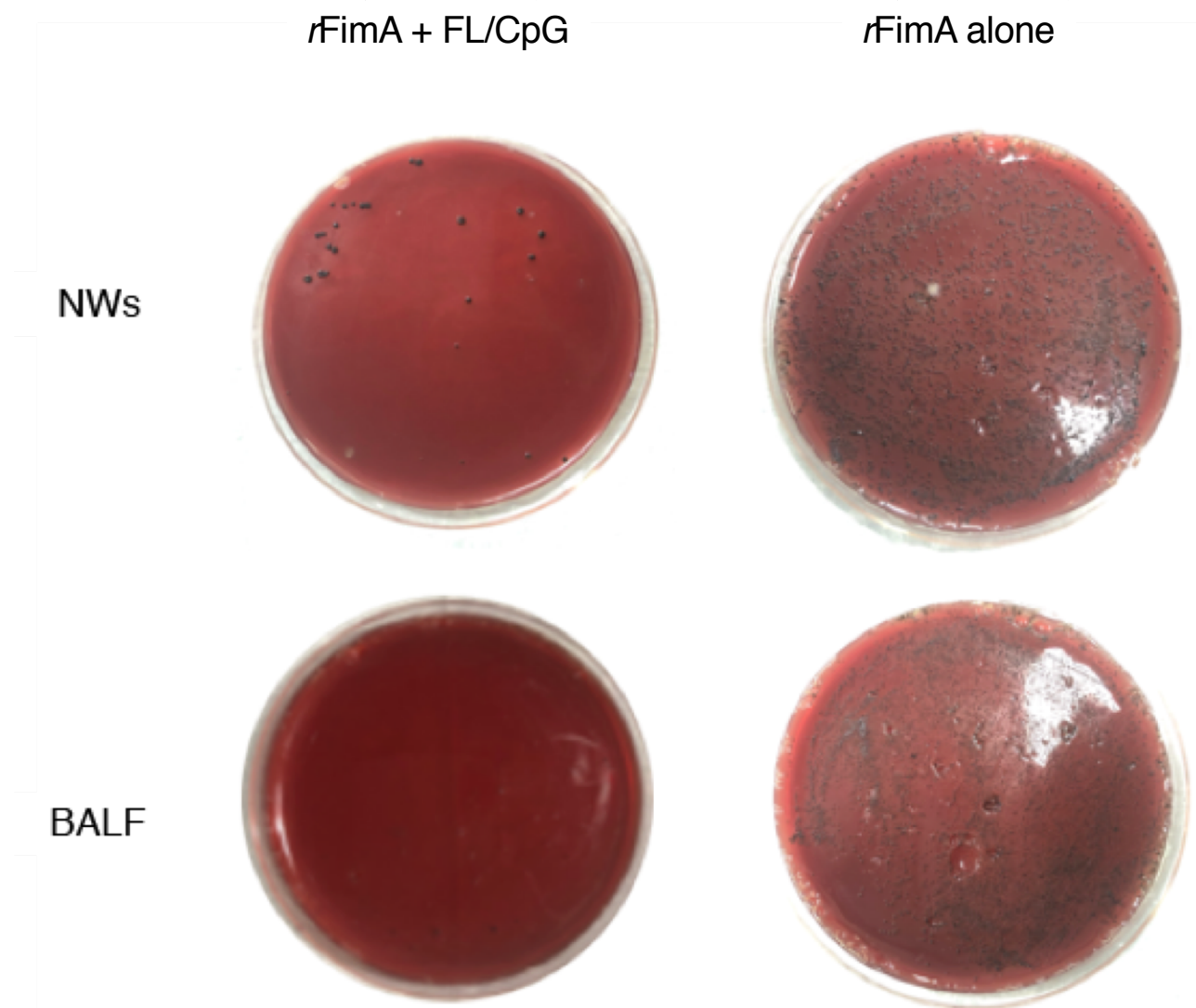

Supplement: Supplementary Figure 1 — Typical FACS plot and gating strategy for NALT and lungs. Mononuclear cells in NALT and lungs were gated by using the forward- and side-scatter properties, and were subsequently analyzed for CD11c+ cells. [file DataSheet_1.pdf]
